# Identification of risk factors and development of predictive models for chronic postsurgical pain after modified radical mastectomy

**DOI:** 10.1080/07853890.2025.2599608

**Published:** 2025-12-07

**Authors:** Ninger Gong, Qinyue Yuan, Pengyan Li, Zhen Zhao, Yi Zhao, Bing Li, Luyao Zhang, Yitian Yang, Jiaqiang Zhang, Wei Zhang

**Affiliations:** a Department of Anesthesiology and Perioperative Medicine, Henan Medical University & Henan Provincial People’s Hospital, Zhengzhou, Henan, China; b Department of Anesthesiology, The First Affiliated Hospital of Henan University of Chinese Medicine, Zhengzhou, Henan, China; c Department of Anesthesiology and Perioperative Medicine, Zhengzhou University People’s Hospital & Henan Provincial People’s Hospital, Zhengzhou, Henan, China; d Department of Anesthesiology and Perioperative Medicine, Henan University People’s Hospital & Henan Provincial People’s Hospital, Zhengzhou, Henan, China

**Keywords:** Breast cancer, modified radical mastectomy, chronic postsurgical pain, prediction model, cohort study

## Abstract

**Objective:**

To investigate the risk factors for chronic postsurgical pain (CPSP) following modified radical mastectomy (MRM) for breast cancer and to establish a clinical prediction model.

**Methods:**

A prospective cohort study was conducted, enrolling patients who underwent MRM for breast cancer. Comprehensive data were systematically collected at three time points: preoperatively, intraoperatively, and postoperatively. Follow-up assessments were performed at 3 and 6 months postoperatively. Separate prediction models were constructed for CPSP at 3 months and 6 months postoperatively. A comprehensive clinical prediction model was developed through a systematic approach.

**Results:**

A total of 235 patients were enrolled in this study. Postoperative insomnia and follicle-stimulating hormone (FSH) levels were independent risk factors for CPSP at both 3 and 6-month postoperatively, while a higher body mass index (BMI) was a protective factor for CPSP at 3-month postoperatively. The AUC values of the model at 3 and 6-month postoperatively were 0.767 (0.706-0.829) and 0.733 (0.666-0.799), respectively. Model performance and stability were further confirmed by receiver operating characteristic (ROC) curves, decision curve analysis (DCA), calibration curves, and a nomogram, indicating good discrimination and clinical applicability.

**Conclusion:**

Postoperative insomnia and FSH levels are independent risk factors for CPSP at 3 and 6-month postoperatively, and a higher BMI is a protective factor for CPSP at 3-month postoperatively. Early identification and management of postoperative insomnia, as well as preoperative screening of FSH levels, may help reduce the risk of CPSP.

## 1. Introduction

Breast cancer is the most prevalent malignancy among women worldwide. The Cancer Statistics 2025 report estimates that, in 2025, breast cancer will account for 15.5% of all new cancer cases among women in the United States [1]. Modified Radical Mastectomy (MRM), despite the increasing adoption of breast reconstruction surgeries, remains a crucial procedure for radical tumor treatment and lymph node dissection, particularly for patients requiring complete tumor resection, where it holds significant clinical value [2].

As defined by the International Association for the Study of Pain (IASP), Chronic Post-Surgical Pain (CPSP) refers to pain persisting for more than 3-month postoperatively in the surgical site and its adjacent areas [3,4]. The incidence of CPSP in patients after breast cancer surgery ranges from 20% to 60%, affecting nearly half of the patient population [5,6]. CPSP not only impairs patients’ quality of life but also frequently coexists with paresthesia, dysfunction, and psychological distress. Its exact mechanism remains unclear, potentially involving intraoperative nerve injury, central sensitization, inflammatory responses, and psychosocial factors [7]. Current conventional interventions include optimizing perioperative analgesia, nerve protection, psychological management, and early rehabilitation guidance; however, their efficacy in treating CPSP remains limited [8,9].

Sex hormones represent a key physiological characteristic of women, however few studies have examined the impact of sex hormone levels on CPSP in this population [10]. Sex hormones such as estrogen and follicle-stimulating hormone (FSH) regulate the central and peripheral nervous systems, influencing pain sensitivity, inflammatory responses, and neural plasticity [11]. Preoperative sex hormone levels also vary among female breast cancer patients. Based on the above analysis, we hypothesize that sex hormones may play a potential role in CPSP, warranting further exploration [12].

CPSP evolves over time. Many studies have assessed CPSP at a single time point, but chronic pain requires long-term evaluation. For instance, one study reported that the incidence of CPSP after cardiac surgery was 29% at 3-month postoperatively and 19% at 6-month postoperatively [13]. Thus, long-term follow-up and predictive models at multiple time points are necessary [14].

Building on existing studies, this study adopts a prospective observational cohort design, incorporating preoperative sex hormone levels (e.g. estrogen, FSH), psychological status, and perioperative factors. Chronic pain was assessed at two postoperative time points (3 and 6 months) to separately develop prediction models for CPSP following modified radical mastectomy. This study aims to identify risk factors for CPSP after modified radical mastectomy for breast cancer, establish and validate a comprehensive prediction tool integrating biological, psychological, and perioperative variables, and provide a basis for preoperative risk assessment and postoperative individualized intervention [15,16].

## 2. Methods

This study protocol was approved by the Ethics Committee of Henan Provincial People’s Hospital [(2025) Ethical Review No. (16)] and registered in the Clinical Trial Registry (ChiCTR2500098431). Written informed consent was obtained from all subjects, and the study was conducted in accordance with the Declaration of Helsinki.

### 2.1. Study design and participants

This is a prospective observational cohort study aimed at developing a prediction model for CPSP after modified radical mastectomy for breast cancer. The study participants were female patients who underwent modified radical mastectomy for breast cancer at Henan Provincial People’s Hospital from January 2025 to September 2025.

Inclusion criteria: (1) aged ≥ 18 years; (2) scheduled to undergo modified radical mastectomy for breast cancer and meeting surgical indications; (3) provided written informed consent and agreed to preoperative psychological status assessment and postoperative pain follow-up; (4) able to complete at least 6-month of postoperative pain assessment follow-up; (5) ASA physical status classification I-III.

Exclusion criteria: (1) pre-existing chronic pain or receiving related treatment before surgery; (2) suffering from severe endocrine diseases (e.g. uncontrolled diabetes); (3) history of intravenous drug or opioid abuse; (4) complicated with severe mental illness or cognitive impairment; (5) pregnant or lactating women; (6) incomplete baseline data.

### 2.2. Data collection and follow-up

Patients were assessed for eligibility upon admission, and written informed consent was obtained from all participants. Data collection was performed by two independent assessors who were blinded to the study protocol. Specifically, one assessor collected preoperative and intraoperative data, and the other was responsible for postoperative data.

Baseline demographic and clinical characteristics, including age, body mass index (BMI), medical comorbidities, menstrual status, medical history, and pathological staging (graded from stage I to IV based on the 8th edition of the American Joint Committee on Cancer Staging System[17]), were collected *via* standardized questionnaires and extracted from electronic medical records (EMR).

Preoperative data were collected on the day before surgery, encompassing psychological and sleep assessments using the Hospital Anxiety and Depression Scale (HADS) and the Pittsburgh Sleep Quality Index (PSQI). The HADS was utilized to evaluate patients’ preoperative psychological status, which is primarily designed for screening anxiety and depressive symptoms among general hospital inpatients [18]. The PSQI was adopted to assess preoperative sleep quality; this scale is suitable for evaluating sleep disorders in patients with sleep or mental conditions, monitoring treatment efficacy, and conducting population-based sleep quality surveys [19]. Additionally, 5-milliliter blood samples were collected for sex hormone detection using the chemiluminescence immunoassay method.

Intraoperative data were retrieved from the EMR, including details such as surgical duration, consumption of opioids and anesthetics, and surgical approaches (e.g. sentinel lymph node biopsy or axillary lymph node dissection). Specific information regarding anesthesia and analgesia administration, including the use of sufentanil, remifentanil, dexamethasone, and penehyclidine, was also documented in detail.

Postoperative data collected included acute and chronic postoperative pain, postoperative insomnia, length of hospital stay, postoperative endocrine therapy and chemotherapy, as well as other relevant data. Postoperative acute pain data were collected at 2 h and 1 day after surgery. Pain intensity was measured using a standardized 11-point Numerical Rating Scale (NRS, 0–10). The collected acute pain data included resting pain at 2 h postoperatively, as well as resting pain and pain during activities at 24 h postoperatively. The same assessor conducted follow-up evaluations at 3 months and 6 months post-surgery to assess the incidence of CPSP, postoperative insomnia, and postoperative menstrual disorder.

### 2.3. Outcomes and definition of CPSP

The primary outcome of this study was to identify the risk factors for CPSP at 3 months postoperatively and develop a corresponding clinical prediction model. The secondary outcome was to identify the risk factors for CPSP at 6 months postoperatively and establish a prediction model specific to this time point.

In the present study, CPSP was diagnosed when patients reported a pain intensity score greater than 0 on the Numerical Rating Scale (NRS, 0–10) during the 3-month and 6-month postoperative follow-up assessments. Specifically, during telephone follow-up, the score of the most severe pain localized in the breast, axilla, or adjacent areas reported by patients over the preceding week was used to determine the occurrence of CPSP.

### 2.4. Statistical analysis

The sample size was calculated based on the EPV (events per variable) principle[20]. Assuming 8 significant risk factors would be analyzed and based on preliminary results, the incidence of postoperative chronic pain was approximately 50%. According to the EPV principle, each risk factor requires at least 10 events. Thus, the minimum number of events required was 80. With a 50% event incidence (based on our preliminary data), the sample size should be 160 patients. Accounting for an expected 30% dropout rate, this study planned to recruit 229 patients. This sample size calculation ensures sufficient statistical power to evaluate the impact of each risk factor while enhancing model stability and reliability.

Data analysis was conducted by an independent person. Our analysis was conducted using univariate analysis to identify potential factors associated with CPSP, using a P-value threshold of 0.1. The Shapiro-Wilk test was used to assess the normality of continuous variables. Normally distributed variables were analyzed using the independent samples t-test, and non-normally distributed variables using the Mann-Whitney U test. Normally distributed data are presented as mean ± standard deviation, counts (percentages), and 95% confidence intervals (95% CI); categorical variables were analyzed using Pearson’s chi-square test. An NRS score >0 was used to define the occurrence of postoperative chronic pain.

Variables with *p* < 0.1 in univariate analysis were selected for collinearity diagnosis using the variance inflation factor (VIF). A VIF < 5 indicated no severe collinearity. LASSO regression was then used for variable shrinkage and screening to control overfitting, variables excluded by LASSO regression were removed from further analysis. Additionally, factors that have been previously identified in the literature as associated with CPSP after breast cancer surgery were also included in the LASSO regression. Selected variables were included in a multivariate logistic regression model to analyze their independent association with CPSP occurrence. All statistical analyses were performed using SPSS version 27.0 and RStudio Team (2025). R was used for advanced variable selection (including LASSO regression) and model validation. A p-value < 0.05 was considered statistically significant.

Model discriminative ability was evaluated using the receiver operating characteristic (ROC) curve and area under the curve (AUC). Goodness-of-fit was assessed using the calibration curve. To enhance model robustness, 10-fold cross-validation was performed. Clinical utility was verified using decision curve analysis (DCA), and a nomogram was constructed for visual presentation. Additionally, we explored the non-linear relationship between FSH levels and the occurrence of CPSP using restricted cubic spline (RCS) models. Through this analysis, we aimed to explore the dose-response relationship between FSH and CPSP. This model was incorporated into the overall model to capture potential non-linear associations that might have been overlooked by traditional linear approaches.

## 3. Results

**Figure 1.**
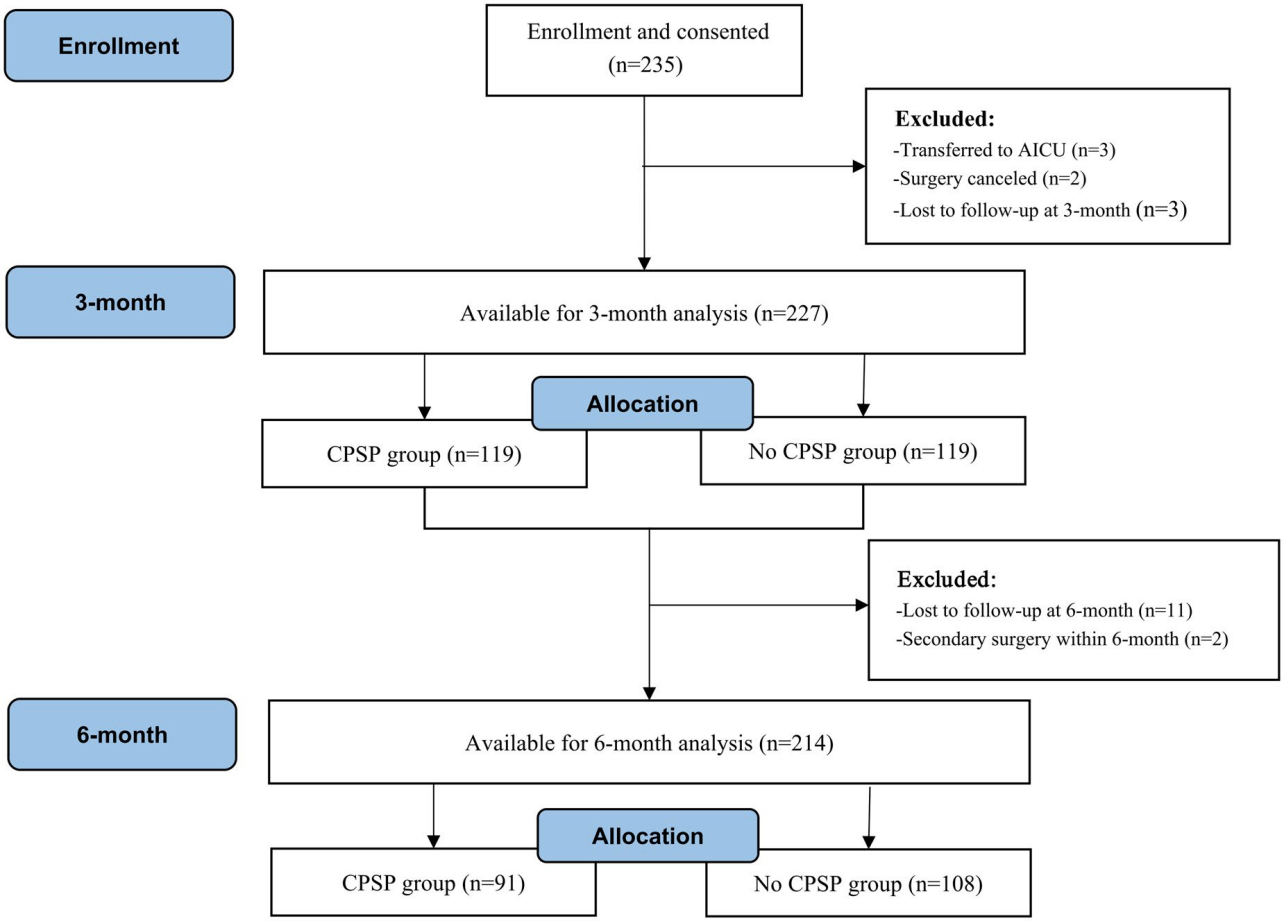
Flow diagram.

The flow chart of this study is shown in Figure 1. Initially, 235 patients were enrolled and provided written informed consent. During follow-up, 3 patients were transferred to the AICU postoperatively, 2 had their surgery canceled, and 3 were lost to follow-up at 3-month, resulting in 227 patients for the 3-month analysis. At 6-month, 11 patients were lost to follow-up and 2 underwent secondary surgery within 6-month, resulting in 214 patients for the final 6-month analysis.

The incidence of CPSP was 52.4% (119/227) at 3-month and 42.5% (91/214) at 6-month. Patients were divided into non-CPSP and CPSP groups based on the presence or absence of CPSP. Significant differences were noted in several baseline characteristics between the CPSP and non-CPSP groups. Compared with the non-CPSP group, patients with CPSP at 3-month (Table 1) showed characteristics of being younger (49.43 years vs. 53.31 years, *p* = 0.002), having a lower BMI (24.1 kg/m^2^ vs. 25.3 kg/m^2^, *p* = 0.009), lower preoperative FSH levels (28.16 IU/L vs. 41.51 IU/L, *p* = 0.001), poorer preoperative sleep quality (a higher proportion of poor/severely poor sleep, *p* < 0.001), and a higher proportion of postoperative insomnia (63.9% vs. 34.9%, *p* < 0.001). A similar trend was observed at 6-month (Table 2), with low FSH, postoperative insomnia, and preoperative sleep disorders remaining significantly associated with CPSP (all *p* < 0.05).

**Table 1.** Demographic and clinical characteristics at 3-month.

Time points	Variable	No CPSP (3-month, *n* = 108)	CPSP (3-month, *n* = 119)	OR (3-month 95% CI)	*p* value (3-month)
Preoperative	Age (years)	53.31 + 8.10	49.43 + 9.70	0.953 (0.925–0.982)	0.002
	BMI (kg.m^-2^)	25.3 + 3.3	24.1 + 3.3	0.90 (0.83–0.97)	0.009
	Education status, n (%)				
	Primary school	31 (28.7%)	26 (21.8%)	1.16 (0.56–2.37)	0.694
	Secondary school	32 (29.6%)	31 (26.1%)	1.36 (0.62–2.94)	0.442
	High school	22 (20.4%)	25 (21.0%)	1.92 (0.92–4.01)	0.083
	College	23 (21.3%)	37 (31.1%)	Reference	Reference
	Menstrual cycle, n (%)				
	Menopause period	61 (67.0%)	46 (53.5%)	Reference	Reference
	Menstrual phase	7 (7.7%)	11 (12.8%)	2.08 (0.75–5.79)	0.159
	Follicular phase	7 (7.7%)	10 (11.6%)	1.89 (0.67–5.35)	0.228
	Ovulatory phase	3 (3.3%)	6 (7.0%)	2.65 (0.63–11.17)	0.184
	Luteal phase	13 (14.3%)	13 (15.1%)	1.33 (0.56–3.13)	0.519
	Medical comorbidities, n (%)				
	Hypertension	22 (20.4%)	18 (15.1%)	0.70 (0.35–1.38)	0.302
	Diabetes mellitus	9 (8.3%)	9 (7.6%)	0.90 (0.34–2.36)	0.830
	Hepatobiliary diseases	2 (1.9%)	6 (5.0%)	2.81 (0.56–14.25)	0.211
	Digestive system	3 (2.8%)	4 (3.4%)	1.22 (0.27–5.57)	0.800
	Gynecological diseases	8 (7.4%)	8 (6.7%)	0.90 (0.33–2.49)	0.841
	Cardiovascular and cerebrovascular diseases	6 (5.6%)	5 (4.2%)	0.75 (0.22–2.52)	0.636
	Respiratory system	2 (1.9%)	2 (1.7%)	0.91 (0.13–6.55)	0.922
	Menopause, n (%)	47 (43.52%)	73 (61.34%)	0.49 (0.29–0.83)	0.008
	Sex hormone				
	Estradiol (pg/mL)	15.0 (15.00, 111.09)	31.8 (15, 98.07)	0.999 (0.996–1.003)	0.742
	Testosterone (ng/mL)	0.26 (0.18, 0.45)	0.30 (0.12, 0.41)	0.68 (0.11–4.37)	0.681
	Progesterone (ng/mL)	0.43 (0.24, 1.14)	0.42 (0.13, 1.28)	1.00 (0.93–1.08)	0.933
	FSH (IU/L)	41.51 (8.32, 69.31)	28.16 (6.92, 46.76)	0.98 (0.97–0.99)	0.001
	LH (IU/L)	22.77 (8.15, 40.39)	15.82 (6.67, 32.48)	1.00 (0.99–1.01)	0.715
	Prolactin (ng/mL)	12.35 (7.63, 20.95)	13.74 (8.04, 24.29)	1.02 (0.99–1.05)	0.304
	Preoperative sleep status, n (%)				
	Good sleep quality	80 (74.8%)	52 (43.7%)	Reference	Reference
	Fair sleep quality	17 (15.9%)	48 (40.3%)	4.34 (2.26–8.36)	<0.001
	Poor sleep quality	8 (7.5%)	15 (12.6%)	2.89 (1.14–7.29)	0.025
	Severe poor sleep quality	2 (1.9%)	4 (3.4%)	3.08 (0.54–17.41)	0.204
	HADS-A, n (%)				
	0	92 (86.0%)	81 (68.1%)	Reference	Reference
	1	13 (12.1%)	29 (24.4%)	2.53 (1.23–5.20)	0.011
	2	2 (1.9%)	8 (6.7%)	4.54 (0.94–22.01)	0.060
	3	0 (0.0%)	1 (0.8%)	–	–
	HADS-D, n (%)				
	0	98 (91.6%)	96 (80.7%)	Reference	Reference
	1	8 (7.5%)	16 (13.4%)	2.04 (0.83–4.99)	0.118
	2	1 (0.9%)	7 (5.9%)	7.15 (0.86–59.18)	0.068
	ASA, n (%)				
	1	11 (10.2%)	17 (14.3%)	Reference	Reference
	2	93 (86.1%)	94 (79.0%)	0.65 (0.29–1.47)	0.305
	3	4 (3.7%)	8 (6.7%)	1.29 (0.31–5.35)	0.722
	Pre-operative chemotherapy, n (%)	15 (13.89%)	23 (19.33%)	1.49 (0.73–3.02)	0.275
	Preoperative biopsy, n (%)	52 (48.1%)	64 (53.8%)	1.25 (0.74–2.11)	0.397
	Preoperative surgery at the surgical site, n (%)	11 (10.2%)	13 (10.9%)	1.08 (0.46–2.53)	0.856
	Preoperative surgery at non-surgical sites, n (%)	17 (15.7%)	19 (16.0%)	1.02 (0.50–2.08)	0.963
	Stage of breast cancer, n (%)				
	0	6 (5.6%)	5 (4.2%)	0.61 (0.19–2.30)	0.516
	I	1 (0.9%)	2 (1.7%)	1.59 (0.14–18.04)	0.710
	IA	19 (17.6%)	9 (7.6%)	0.38 (0.16–0.91)	0.030
	IB	1 (0.9%)	1 (0.8%)	0.79 (0.05–13.02)	0.871
	IIA	15(13.9%)	17 (14.3%)	0.90 (0.41–2.00)	0.973
	IIB	10 (9.3%)	12 (10.1%)	0.95 (0.38–2.40)	0.916
	IIIA	2 (1.9%)	9 (7.6%)	3.57 (0.74–17.33)	0.115
	IIIB	1 (0.9%)	0 (0.0%)	0.00	1.000
	IIIC	7 (6.5%)	5(4.2%)	0.57 (0.17–1.90)	0.358
	IV	0 (0.0%)	1 (0.8%)	–	1.000
Intraoperative	Duration of surgery (min)	125.0 (100, 168)	130.0 (100, 165)	1.00 (1.00-1.00)	1.000
	Sufentanil (μg)	30.0(20.0, 30.0)	30.0 (20.0, 30.0)	1.03 (1.00–1.07)	0.080
	Remifentanil (mg)	0.48 (0.36, 0.60)	0.50 (0.36, 0.70)	0.94 (0.41–2.15)	0.881
	Dexamethasone (mg)	5.0 (5.00, 5.00)	5.0 (5.00, 5.00)	0.908	0.341
	Penehyclidine (mg)	0.5 (0.50, 0.50)	0.5 (0.50, 0.50)	0.42 (0.07–2.66)	0.353
	Airway devices, n (%)			2.800	0.094
	Intubation	18 (16.7%)	11 (9.2%)	Reference	Reference
	Glottis mask airway	90 (83.3%)	108 (90.8%)	0.51 (0.23–1.13)	0.099
	Location of surgery, n (%)				
	Bilateral	2 (1.9%)	3 (2.6%)	Reference	Reference
	Right Left	57 (54.8%) 45 (43.3%)	56 (47.9%) 58 (49.6%)	1.16 (0.19–7.26) 0.76 (0.45–1.30)	0.871 0.321
	Laparoscope, n (%)	4 (3.7%)	8 (6.7%)	1.87 (0.55–6.41)	0.317
	Breast conservation, n (%)	15 (13.9%)	30 (25.2%)	2.09 (1.05–4.14)	0.035
	Axillary preservation, n (%)	46 (42.6%)	43 (36.1%)	0.76 (0.45–1.30)	0.440
	Lymph node dissection, n (%)	73 (67.6%)	79 (66.4%)	0.95 (0.54–1.65)	0.847
	Lymph node excision, n (%)	15 (13.9%)	21 (17.6%)	1.33 (0.65–2.73)	0.440
	Length of PACU stay (min)	60.0 (50, 80)	60.0 (48.75, 80.00)	1.00 (0.99–1.01)	0.636
Postoperative	Postoperative pain score (score)				
	24h during activities	2 (2, 2)	2 (2, 2)	1.07 (0.54–2.11)	0.841
	24h at rest	1 (1, 1)	1 (1, 1)	1.26 (0.47–3.40)	0.646
	2h at rest	2 (0, 3)	2 (1, 3)	1.01 (0.82–1.25)	0.918
	Postoperative analgesia pump, n (%)	90 (83.3%)	103 (86.6%)	1.29 (0.62–2.67)	0.498
	Length of hospital stay (days)	10 (8, 12)	10 (8, 13)	1.03 (0.95–1.12)	0.425
	Postoperative chemotherapy, n (%)	69 (63.9%)	63 (52.9%)	0.64 (0.37–1.08)	0.096
	Postoperative endocrine therapy, n (%)	16 (14.8%)	21 (17.6%)	1.23 (0.61–2.51)	0.564
	Postoperative targeted therapy, n (%)	15 (13.9%)	16 (13.4%)	0.96 (0.45–2.06)	0.923
	Postoperative insomnia, n (%)	37 (34.9%)	76 (63.9%)	3.30 (1.91–5.70)	<0.001
	Menstrual disorder, n (%)	26 (24.5%)	40 (33.61%)	0.829 (0.61–1.12)	0.223

Data are reported as Mean ± SD, median (IQR), or number (%) as appropriate. Abbreviations: ASA, American Society of Anesthesiologists; BMI, Body mass index; HADS, Hospital Anxiety and Depression Scale; PACU, post-anesthesia care unit.

**Table 2.** Demographic and clinical characteristics at 6-month.

Time Points	Variable	No CPSP (6-month, *n* = 123)	CPSP (6-month, *n* = 91)	OR (6-month 95% CI)	*p value* (6-month)
Preoperative	Age (years)	52.20 + 8.6	50.91 + 9.4	0.98 (0.96–1.01)	0.301
	BMI (kg.m^-2^)	24.65 + 3.2	24.81 + 3.6	1.01 (0.94–1.10)	0.729
	Education status, n (%)				0.076
	Primary school	33 (26.8%)	22 (24.2%)	Reference	Reference
	Secondary school	39 (31.7%)	20 (22.0%)	0.77 (0.36–1.65)	0.500
	High school	28 (22.8%)	18 (19.8%)	0.96 (0.43–2.15)	0.929
	College	23 (18.7%)	31 (34.1%)	2.02 (0.94–4.34)	0.071
	Menstrual cycle, n (%)				0.097
	Menopause period	65 (63.7%)	38 (55.9%)	Reference	Reference
	Menstrual phase	10 (9.8%)	7 (10.3%)	1.18 (0.42–3.41)	0.736
	Follicular phase	7 (6.9%)	9 (13.2%)	2.20 (0.76–6.38)	0.147
	Ovulatory phase	2 (2.0%)	7 (10.3%)	5.99 (1.18–30.30)	0.031
	Luteal phase	18 (17.6%)	7 (10.3%)	0.67 (0.26–1.74)	0.405
	Medical comorbidities, n (%)				
	Hypertension	22 (17.9%)	17 (18.7%)	1.06 (0.52–2.13)	0.882
	Diabetes mellitus	12 (9.8%)	4 (4.4%)	0.43 (0.13–1.37)	0.151
	Hepatobiliary diseases	0 (0.0%)	1 (1.1%)	3.52 (0.67–18.55)	0.138
	Digestive system	2 (1.6%)	5 (5.5%)	3.52 (0.67–18.55)	0.138
	Gynecological diseases	8 (6.5%)	6 (6.6%)	1.02 (0.34–3.03)	0.979
	Cardiovascular and cerebrovascular diseases	5 (4.1%)	6 (6.6%)	1.67 (0.49–5.64)	0.412
	Respiratory system	1 (0.8%)	2 (2.2%)	0.44 (0.05-4.34)	0.486
	Menopause, n (%)	65 (52.8%)	38 (41.8%)	0.64 (0.37–1.11)	0.109
	Sex hormone				
	Estradiol (pg/mL)	15.6 (15.0, 86.6)	25.7 (15.0, 118.7)	1.00 (1.00-1.00)	0.888
	Testosterone (ng/mL)	0.29 (0.18, 0.40)	0.30 (0.15, 0.45)	1.29 (1.19–8.71)	0.793
	Progesterone (ng/mL)	0.39 (0.21, 1.23)	0.49 (0.25, 1.24)	1.0 (0.94–1.08)	0.840
	FSH (IU/L)	40.26 (8.26, 66.59)	128.08 (6.92, 45.11)	0.98 (0.97–0.99)	0.003
	LH (IU/L)	24.69 (8.16, 41.44)	15.79 (7.04, 31.08)	1.00 (0.99–1.01)	0.797
	Prolactin (ng/mL)	13.74 (7.63, 22.34)	12.04 (8.04, 21.66)	1.01 (0.97–1.04)	0.766
	Preoperative sleep status, n (%)				0.003
	Good sleep quality	86 (69.9%)	41 (45.1%)	Reference	Reference
	Fair sleep quality	26 (21.1%)	37 (40.7%)	2.99 (1.60-5.57)	0.001
	Poor sleep quality	8 (6.5%)	11 (12.1%)	2.88 (1.08-7.71)	0.035
	Severe poor sleep quality	3 (2.4%)	2 (2.2%)	1.40 (0.23–8.70)	0.719
	HADS-A, n (%)				0.028
	0	104 (84.6%)	62 (68.1%)	Reference	Reference
	1	17 (13.8%)	23 (25.3%)	2.27 (1.13–4.58)	0.022
	2	1 (0.8%)	6 (6.6%)	10.97 (1.18–85.56)	0.034
	3	1 (0.8%)	0 (0.0%)	–	1.00
	HADS-D, n (%)				0.078
	0	113 (91.9%)	74 (81.3%)	Reference	Reference
	1	7 (5.7%)	13 (14.3%)	2.84 (1.08–7.44)	0.034
	2	3 (2.4%)	4 (4.4%)	2.04 (0.44–9.36)	0.361
	ASA, n (%)				0.111
	1	13 (10.6%)	12 (13.2%)	Reference	Reference
	2	107 (87.0%)	71 (78.0%)	0.72 (0.31–1.67)	0.441
	3	3 (2.4%)	8 (8.8%)	2.89 (0.62–13.50)	0.177
	Pre-operative chemotherapy, n (%)	16 (13.0%)	17 (18.7%)	1.54 (0.73–3.23)	0.258
	Preoperative biopsy, n (%)	65 (52.8%)	48 (52.7%)	1.00 (0.58–1.71)	0.989
	Preoperative surgery at the surgical site, n (%)	12 (9.8%)	10 (11.0%)	1.14 (0.47–2.77)	0.769
	Preoperative surgery at non-surgical sites, n (%)	17 (13.8%)	16 (17.6%)	1.33(0.63–2.80)	0.452
	Stage of breast cancer, n (%)				0.738
	0	4 (3.3%)	6 (6.6%)	1.92(0.51–7.23)	0.336
	I	1 (0.8%)	2 (2.2%)	2.56 (0.22–29.16)	0.449
	IA	19 (15.4%)	8 (8.8%)	0.54 (0.22–1.35)	0.186
	IB	1 (0.8%)	1 (1.1%)	1.28 (0.08–21.04)	0.863
	IIA	18 (14.6%)	13 (14.3%)	0.92 (0.41–2.09)	0.849
	IIB	15 (12.2%)	7 (7.7%)	0.60 (0.22–1.59)	0.303
	IIIA	4 (3.3%)	5 (5.5%)	1.60 (0.41–6.32)	0.503
	IIIB				
	IIIC	5 (4.1%)	6 (6.6%)	1.54 (0.44–5.37)	0.502
	IV	1 (0.8%)	0 (0.0%)	–	1.000
Intraoperative	Duration of surgery (min)	137.28 (62.1)	140.77 (54.4)	1.00 (1.00-1.00)	0.669
	Sufentanil (μg)	30.0 (20.0, 30.0)	30.0 (20.0, 30.0)	1.02 (0.98–1.06)	0.308
	Remifentanil (mg)	0.5 (0.36, 0.60)	0.5 (0.36, 0.70)	1.137 (0.48–2.70)	0.770
	Dexamethasone (mg)	5.0 (5.00, 5.00)	5.0 (5.00, 5.00)	0.74	0.029
	Penehyclidine (mg)	0.5 (0.50, 0.50)	0.5 (0.50, 0.50)	0.54 (0.08–3.62)	0.529
	Airway devices, n (%)				
	Intubation	103 (83.7%)	84 (92.3%)	Reference	Reference
	Glottis mask airway	20 (16.3%)	7 (7.7%)	0.43 (0.17–1.06)	0.068
	Location of surgery, n (%)				0.604
	Bilateral	3 (2.5%)	2 (2.3%)	Reference	Reference
	Right Left	57 (47.5%) 60 (50.0%)	48 (54.5%) 38 (43.2%)	1.26 (0.20–7.87) 0.95 (1.52–5.95)	0.802 0.956
	Laparoscope, n (%)	8 (6.5%)	2 (2.2%)	0.32 (0.07–1.56)	0.159
	Breast conservation, n (%)	23 (18.7%)	17 (18.7%)	1.00 (0.50–2.00)	0.997
	Axillary preservation, n (%)	49 (39.8%)	35 (38.5%)	0.94 (0.54–1.65)	0.839
	Lymph node dissection, n (%)	80 (65.0%)	64 (70.3%)	1.27 (0.71–2.28)	0.415
	Lymph node excision, n (%)	18 (14.6%)	16 (17.6%)	1.24 (0.60–2.60)	0.560
	Length of PACU stay (min)	68.98 (27.0)	63.33 (24.2)	0.99 (0.98–1.00)	0.120
Postoperative	Postoperative pain score				
	24 h during activities	2 (2, 2)	2 (2, 2)	1.03 (0.52–2.04)	0.943
	24h at rest	1 (1, 1)	1 (1,1)	1.39 (0.50–3.91)	0.531
	2h at rest	2 (0, 3)	2 (1, 3)	1.11 (0.89–1.38)	0.344
	Postoperative analgesia pump, n (%)	99 (80.5%)	83 (91.2%)	2.52 (1.07–5.89)	0.034
	Length of hospital stay (days)	10.0 (8.00, 12.75)	11.0 (8.00, 12.00)	1.02 (0.94–1.11)	0.585
	Postoperative chemotherapy, n (%)	76 (61.8%)	49 (53.8%)	0.72 (0.42–1.25)	0.244
	Postoperative endocrine therapy, n (%)	18 (14.6%)	16 (17.6%)	1.24 (0.60–2.60)	0.560
	Postoperative targeted therapy, n (%)	17 (13.8%)	11 (12.1%)	0.86 (0.38–1.93)	0.710
	Postoperative insomnia, n (%)	45 (36.6%)	59 (64.8%)	3.20 (1.82–5.63)	<0.001
	Menstrual disorder, n (%)	27 (22.0%)	34 (37.4%)	2.12 (1.16–3.87)	0.014

Data are reported as Mean ± SD, median (IQR), or number (%) as appropriate. Abbreviations: ASA, American Society of Anesthesiologists; BMI, body mass index; HADS, Hospital Anxiety and Depression Scale; PACU, post-anesthesia care unit.

LASSO regression was applied for automatic variable screening and shrinkage to reduce interference from redundant variables and overfitting risk. LASSO regression determined the optimal regularization parameter (alpha) *via* 10-fold cross-validation, ultimately retaining 5 variables closely related to 3-month CPSP: age, BMI, menopausal status, preoperative FSH level, PSQI score (preoperative sleep quality), and postoperative insomnia status. Among these, age (LASSO coefficient = −0.0258) and BMI (LASSO coefficient = −0.0300) were negatively correlated, suggesting potential protective effects within a certain range; the PSQI score (LASSO coefficient = +0.0314) was positively correlated, reflecting the potential impact of sleep factors on CPSP. For 6-month CPSP, LASSO retained 5 key variables: PSQI score, postoperative insomnia, HADS-A, education status and preoperative FSH level. The LASSO coefficient for postoperative insomnia was +0.080, indicating a strong positive risk factor; the FSH level coefficient was −0.014, suggesting a potential protective effect.

After univariate analysis, collinearity diagnosis, and LASSO variable screening, selected variables were included in a multivariate binary logistic regression model to further clarify their independent impact on CPSP occurrence.

Variables included in the 3-month CPSP multivariate analysis (Table 3) were age, BMI, menopausal status, preoperative PSQI score, and postoperative insomnia. Results showed that postoperative insomnia was an independent risk factor for CPSP (OR = 2.63, 95%CI: 1.22–5.66, *p* = 0.014), indicating a significantly increased risk in patients with postoperative insomnia; BMI was a protective factor (OR = 0.87, 95%CI: 0.79–0.96, *p* = 0.007), with each 1-unit increase in BMI associated with a 13% reduction in CPSP risk; preoperative FSH level was also a protective factor (OR = 0.98, 95% CI: 0.96–0.99, *p* = 0.001).

**Table 3.** Multivariable logistic regression for 3 and 6-month outcomes.

Variable (3-month)	OR (3-month, 95% CI)	*p* value (3-month)	Variable (6-month)	OR (6-month, 95% CI)	*p* value (6-month)
FSH (IU/L)	0.98 (0.96, 0.99)	0.001	FSH (IU/L)	0.98 (0.97, 0.99)	0.003
Postoperative insomnia	2.63 (1.22, 5.66)	0.014	Postoperative insomnia	2.42 (1.14, 5.16)	0.022
Preoperative sleep status			Preoperative sleep status		
Fair sleep quality	3.08 (1.36, 6.98)	0.007	Fair sleep quality	1.59 (0.65, 3.86)	0.311
Poor sleep quality	1.49 (0.48, 4.62)	0.395	Poor sleep quality	1.42 (0.37, 5.49)	0.604
Severe poor sleep quality	1.48 (0.22, 9.90)	0.684	Severe poor sleep quality	0.54 (0.06, 5.10)	0.598
Age (years)	0.97 (0.919, 1.018)	0.199	HADS-A		
BMI (kg.m^-2^)	0.87 (0.79–0.96)	0.007	1	1.17 (0.46, 2.99)	0.744
Menopause	2.41 (0.87,6.70)	0.093	2	6.78 (0.65, 70.97)	0.099
			3		0.988
			Education Status 4	1.72 (0.87, 3.42)	0.120

Estimates of the odds ratios with corresponding Wald 95% CIs. CI, confidence interval; OR, odds ratio; Education Status 4: Refers to an education level of ‘College’, with reference to the baseline of ‘Primary school’.

The 6-month CPSP multivariate model (Table 3) included PSQI score, postoperative insomnia, HADS-A score, education status and preoperative FSH level. Results showed that postoperative insomnia was a significant independent risk factor (OR = 2.42, 95%CI: 1.14–5.16, *p* = 0.022), with a significantly increased CPSP risk; FSH level was a protective factor (OR = 0.98, 95% CI: 0.97–0.99, *p* = 0.003), with each 1 IU/L increase in FSH associated with approximately a 1% reduction in CPSP risk.

Model performance was comprehensively validated across four dimensions: discriminative ability, goodness-of-fit, stability, and clinical utility, covering both 3 and 6-month time points.

**Figure 2.**
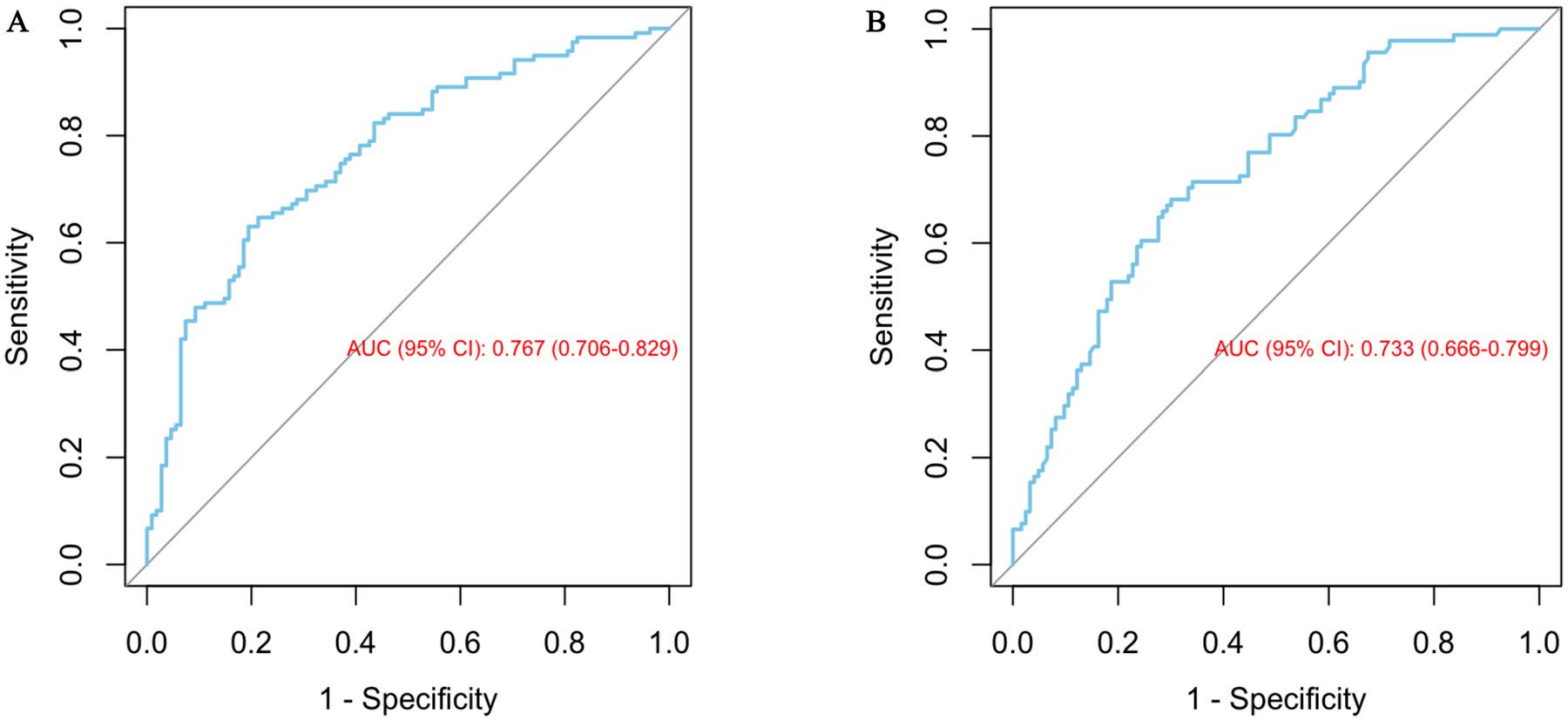
Receive operating characteristic (ROC) curve for evaluating the diagnostic performance of 3-month model and 6-month model. Receiver operating characteristic (ROC) curves for the chronic postsurgical pain (CPSP) prediction models at 3-month (A) and 6-month (B) postoperatively are presented. Panel A illustrates the ROC curve of the 3-month model with an area under the curve (AUC) of 0.767 (0.706–0.829), whereas Panel B demonstrates that of the 6-month model with an AUC of 0.733 (0.666–0.799).

First, discriminative ability was evaluated using the ROC curve and AUC (Figure 2). The 3-month model AUC was 0.767 (0.706–0.829) (Figure 2A), and the 6-month model AUC was 0.733 (0.666–0.799) (Figure 2B), both indicating good predictive performance. Using the common criterion (AUC > 0.70 for acceptable discriminative ability), both models effectively distinguished high-risk from low-risk patients. Notably, although the 6-month model AUC was slightly lower, it remained within a reasonable range, demonstrating temporal extensibility of predictive performance.

To further verify model robustness and generalizability, 10-fold cross-validation was used for internal validation. The full dataset was equally divided into 10 subsets; in each round, 9 subsets were used for training and 1 for validation, until all subsets had served as the validation set. This method effectively reduces sample bias and evaluates model generalizability to new samples. Results showed cross-validated AUC ranges of 0.755 ± 0.138 for the 3-month model and 0.706 ± 0.092 for the 6-month model, with small overall fluctuations, indicating good stability and reduced overfitting risk.

**Figure 3.**
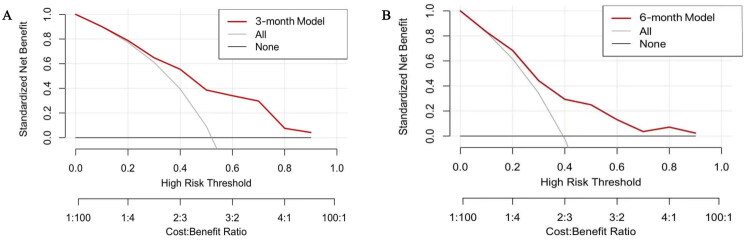
Decision curve analysis (DCA) comparing the clinical utility of the CPSP prediction models at 3 and 6-month. Decision curve analysis (DCA) comparing the clinical utility of the CPSP prediction models at 3 and 6-month postoperatively. The red line represents the 3-month model (A), and the 6-month model (B). The gray line indicates a “treat all” strategy, and the black line indicates a “treat none” strategy.

Clinical utility was evaluated using Decision Curve Analysis (DCA) (Figure 3), which assesses the net benefit of the model at different risk thresholds to measure its practical clinical value. The 3-month model demonstrated consistently higher net benefit across a broader range of threshold probabilities (0.05–0.65) (Figure 3A), while the 6-month model showed benefit within a narrower range (0.05–0.50) and with generally lower values (Figure 3B). These findings suggest that the earlier prediction model may offer greater utility for guiding intervention decisions, particularly in settings where lower or moderate risk thresholds are applied.

**Figure 4.**
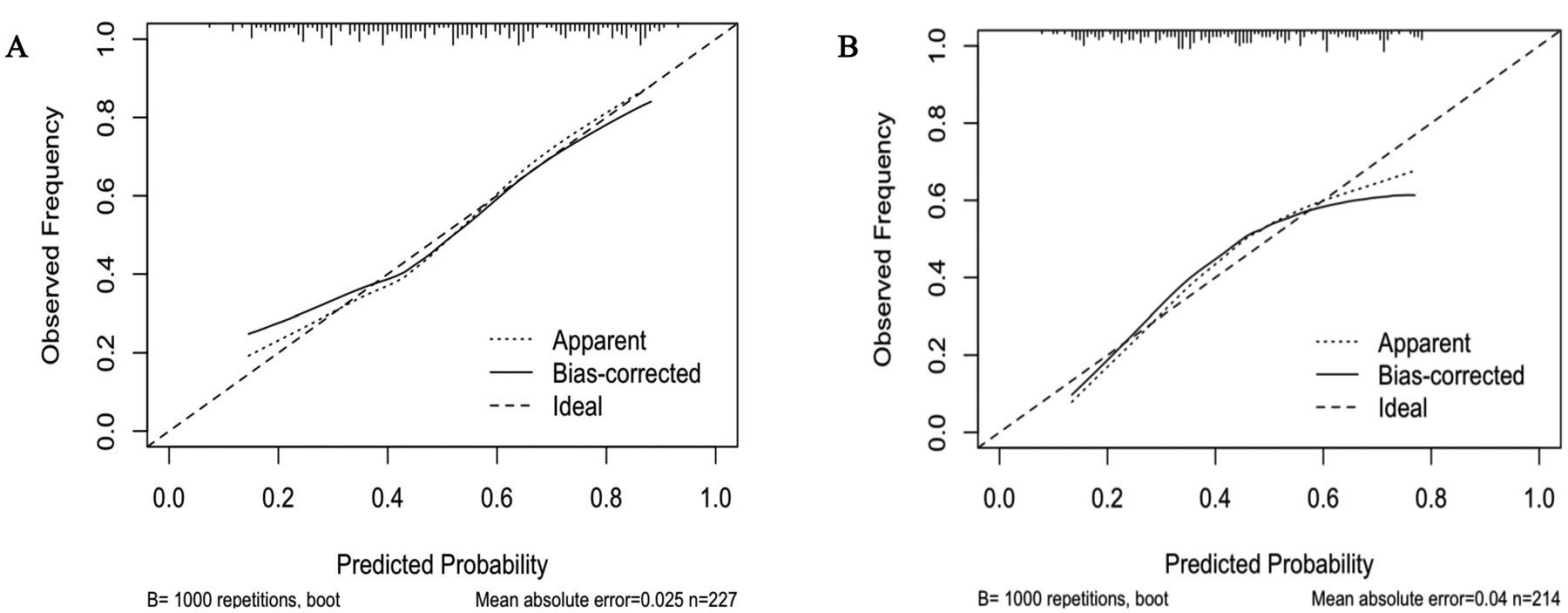
Calibration curves of the CPSP prediction models: comparison of observed and predicted probabilities. Calibration curves for the chronic postsurgical pain (CPSP) prediction models at 3-month (A) and 6-month (B) after surgery are depicted. The dashed line denotes perfect calibration, where predicted probabilities align with observed outcomes. The dotted line (labeled “Apparent”) reflects the model performance on the original dataset, whereas the solid line (labeled “Bias-corrected”) illustrates the performance following 1000 bootstrap repetitions. The mean absolute error was 0.025 for the 3-month model and 0.04 for the 6-month model, indicating good calibration overall, with the 3-month model exhibiting slightly superior performance.

To assess calibration performance, calibration curves were plotted for both the 3-month and 6-month CPSP prediction models (Figure 4). The 3-month model demonstrated a mean absolute error of 0.025, indicating strong agreement between predicted and observed probabilities (Figure 4A). The 6-month model showed a mean absolute error of 0.04, reflecting acceptable calibration performance, albeit slightly inferior to the 3-month model (Figure 4B). In both curves, the bias-corrected line (after 1000 bootstrap repetitions) closely approximated the ideal reference line, supporting the reliability of the model predictions.

**Figure A1.**
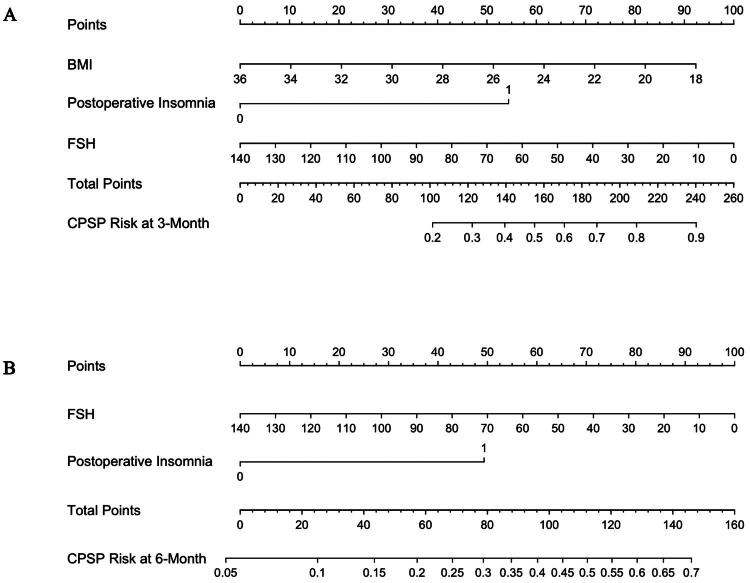
Nomograms for predicting the probability of CPSP. Nomograms for predicting the probability of chronic postsurgical pain (CPSP) at 3-month (A) and 6-month (B) postoperatively are presented. The 3-month model incorporates follicle-stimulating hormone (FSH), postoperative insomnia, and body mass index (BMI), whereas the 6-month model includes FSH and postoperative insomnia. Each predictor corresponds to a specific point value on the “Points” axis. The total points can be summed to estimate an individual’s probability of developing CPSP using the scale at the bottom.

Finally, to facilitate rapid clinical assessment of patients’ CPSP risk, visual nomograms were constructed based on the multivariate logistic regression models (Figure A1). The 3-month model included BMI, FSH level, and postoperative insomnia (Figure A1(A)); the 6-month model included FSH level and postoperative insomnia (Figure A1(B)). By assigning scores to each variable and converting total scores to predicted probabilities, individualized quantitative assessment of preoperative CPSP risk can be achieved, enabling rapid bedside interpretation and clinical prediction of chronic postsurgical pain.

**Figure A2.**
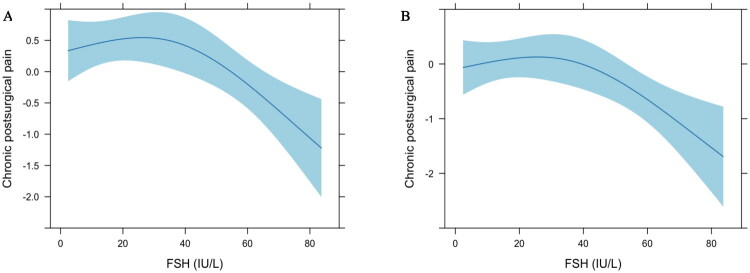
Restricted cubic spline (RCS) curves for analyzing the dose-response relationship between FSH and chronic postsurgical pain risk. Restricted cubic spline (RCS) curves illustrating the association between follicle-stimulating hormone (FSH) levels and the risk of chronic postsurgical pain (CPSP) at 3-month (A) and 6-month (B) after surgery are presented. The shaded area denotes the 95% confidence interval. A nonlinear inverse relationship was identified, whereby higher FSH levels were associated with a lower risk of CPSP.

At 3 and 6-month follow-up after MRM, we examined the association between follicle-stimulating hormone (FSH) levels and the risk of CPSP using restricted cubic spline (RCS) models, adjusting for potential confounders including age, body mass index, menopausal status, adjuvant therapy, and baseline pain scores (Figure A2). A nonlinear relationship was observed between FSH levels and CPSP risk at 3-month (Figure A2(A)). CPSP risk remained relatively stable or slightly increased at low-to-moderate FSH levels, but gradually decreased with higher FSH levels. These findings suggest that FSH levels may influence the occurrence of CPSP within a certain range. The nonlinear association between FSH levels and CPSP risk persisted and became more pronounced at 6-month (Figure A2(B)). CPSP risk showed little change at low-to-moderate FSH levels, but declined significantly at higher FSH levels. Compared with the 3-month results, the effect of FSH on CPSP was more evident at 6-month. All models were constructed using the RCS method. Solid lines represent adjusted odds ratios (ORs), and shaded areas indicate 95% confidence intervals. The models demonstrated good fit, and the associations between FSH levels and CPSP risk were statistically significant at both time points (*p* < 0.05).

## 4. Discussion

This study identified several significant factors associated with CPSP following breast cancer surgery, including postoperative insomnia, FSH levels, and BMI. Specifically, lower preoperative FSH levels were significantly correlated with an increased risk of CPSP at both 3 and 6 months postoperatively. This finding suggests that FSH may exert a protective effect against the development of CPSP. Additionally, postoperative insomnia was consistently linked to a higher risk of CPSP, highlighting the importance of addressing sleep disturbances in the management of chronic pain. By contrast, BMI was associated with a reduced risk of CPSP at 3 months; however, this association did not persist at the 6-month follow-up. Taken together, these findings support a multidimensional model of CPSP that integrates endocrine, psychological, and somatic factors, and underscore the existence of potentially modifiable intervention targets-particularly sleep disturbances-for mitigating CPSP risk in clinical practice.

Postoperative insomnia emerged as a significant risk factor for CPSP in breast cancer patients, with consistent associations observed at both 3 and 6 months post-surgery. Previous studies have shown that poor preoperative sleep quality and disrupted sleep continuity may exacerbate the intensity of acute postoperative pain [21], and poor sleep quality increases the risk of chronic pain [22]. This finding may be related to the bidirectional interaction between sleep and pain: insufficient sleep enhances pain perception, while persistent pain disrupts sleep quality [23]. Thus, identifying and intervening in postoperative insomnia may become a key strategy to reduce the incidence of CPSP. Improving sleep quality-*via* interventions such as cognitive-behavioral therapy or short-term medication-may significantly lower the incidence of postoperative chronic pain. Our results are consistent with this body of evidence and further emphasize the central role of psychological and sleep-related factors in the transition from acute to chronic pain.

FSH, secreted by the anterior pituitary gland, plays a pivotal role in regulating the menstrual cycle and ovarian function. Beyond its reproductive effects, recent studies have implicated FSH in the occurrence and perception of pain. In the context of chronic non-cancer pain, a review investigating FSH’s role across various conditions revealed that alterations in FSH levels may modulate pain severity-particularly in female patients-potentially by regulating nervous system sensitivity and pain thresholds [24]. Emerging literature suggests that gender differences in pain perception and the underlying mechanisms of CPSP may be partially mediated by the effects of sex hormones (including FSH) on pain processing pathways. Specifically, protein kinase A signaling, which contributes to gender differences in chronic pain, may be regulated by FSH, thereby influencing the persistence and severity of pain [25]. In perimenopausal women, fluctuations in FSH levels are likely closely associated with pain perception [26]. Another study demonstrated that FSH-induced depressive-like behaviors, upregulated expression of inflammatory factors, and impaired synaptic plasticity in mice could be attenuated by downregulating the expression of FSH receptors in the hippocampus [27]. Collectively, these findings indicate that FSH, as a key sex hormone, is closely associated with pain perception in the human body; however, current research on its regulatory mechanisms remains insufficiently in-depth. Our study further supports the potential protective role of FSH in chronic pain, suggesting that preoperative FSH levels could serve as a predictive biomarker for assessing the risk of postoperative CPSP. The specific underlying mechanisms, therefore, require further investigation.

BMI was identified as a protective factor for CPSP at 3 months post-surgery, with higher BMI associated with a lower risk of chronic pain; however, this relationship was not sustained at 6 months. This suggests that the impact of BMI on CPSP may vary over time, and other factors may become more influential in the long term. The inconsistent relationship between BMI and CPSP at different follow-up time points may be attributed to changes in the underlying mechanisms of pain. In the early postoperative period, higher BMI may exert a protective effect against pain, potentially through mechanical or hormonal pathways that modulate pain signaling. However, as patients progress further into the recovery phase, other factors-such as psychological distress, inflammation [14],and central sensitization-may have a more prominent impact on the development of CPSP. The conflicting findings regarding the BMI-CPSP relationship in existing literature may reflect differences in study design, study populations, and the specific mechanisms under investigation. Some studies report a protective effect of higher BMI on CPSP, while others find no association or even a positive correlation between BMI and chronic pain [28]. These inconsistencies highlight the complexity of the relationship between BMI and CPSP, indicating that further research is required to explore how BMI interacts with other risk factors in the pathogenesis of CPSP.

In this study, CPSP was defined as persistent pain for at least 3 months after surgery, with a NRS score greater than 0. This threshold was selected based on several considerations. First, postoperative pain scores-both on the first postoperative day and during follow-up-were generally low. Nevertheless, even mild, persistent pain can significantly impair patients’ quality of life and may signal the early stages of chronic pain. Second, using an NRS score > 0 allows for the inclusion of all patients with persistent pain, including those with mild but ongoing pain, who might otherwise be overlooked in clinical practice. Third, in previous studies, some have adopted higher thresholds (e.g. NRS ≥ 3) to define clinically significant CPSP, while others have used the threshold of NRS > 0 and demonstrated that even low-intensity pain can adversely affect quality of life [20,29,30]. This threshold is employed in chronic pain research to encompass a broader spectrum of pain experiences. However, we acknowledge that the use of NRS > 0 may also include patients with very mild symptoms, which could potentially overestimate the clinical burden of CPSP in certain contexts.

To the best of our knowledge, our provide new insights for early CPSP prediction and intervention, particularly regarding postoperative insomnia management and preoperative sex hormone predictive value. Routine screening for postoperative insomnia, early sleep intervention (e.g. cognitive-behavioral therapy or short-term medication) for affected patients, and integrating sleep quality assessment into CPSP management may reduce incidence. Based on our results, we recommend preoperative FSH testing in this population to facilitate early CPSP prediction.

Another key strength of this study resides in its direct clinical applicability. The nomograms developed based on our predictive models provide an intuitive and practical tool for individualized CPSP risk assessment. By integrating readily accessible variables-including FSH levels, BMI, and postoperative insomnia-the nomograms support two pivotal clinical applications. First, they facilitate preoperative counseling, enabling clinicians to communicate personalized CPSP risk profiles to patients. Second, they allow for the early identification of high-risk patients, laying a foundation for the implementation of intensified analgesic and psychological interventions. These tools hold the potential to optimize precision perioperative care and facilitate earlier, more targeted interventions aimed at reducing the incidence of persistent postoperative pain.

Our study provides valuable insights into the prediction of CPSP and offers a practical tool for predicting CPSP in patients with breast cancer. However, several limitations of this study should be acknowledged. First, it did not distinguish between different subtypes of CPSP (e.g. neuropathic pain versus musculoskeletal pain). Previous literature has indicated that the underlying mechanisms of neuropathic pain differ significantly from those of other pain types, which requires distinct treatment regimens. Therefore, future studies should identify neuropathic pain as a separate entity to explore its specific relationship with the development of CPSP. Second, no distinction was made between chronic resting pain and pain during activities. The mechanisms underlying these two types of pain are distinct: resting pain may be primarily mediated by neurological pathways, whereas pain during activities is more closely associated with muscle and joint loading. The failure to differentiate between them may limit the comprehensive assessment of chronic pain and hinder a deeper understanding of its underlying mechanisms. Third, as a single-center study with a relatively homogeneous sample, the generalizability of our findings may be limited. Multi-center, large-sample studies are therefore needed to validate the external validity of our results. Finally, variable selection in this study was primarily based on a data-driven approach. While this strategy effectively identified risk factors with statistical significance, and although we incorporated several variables that lacked statistical significance but have been previously reported to be potentially associated with CPSP in existing literature, it may still overlook certain clinically important variables-ones that, despite having no significant statistical correlation, may be relevant to the development of CPSP.

## 5. Conclusion

Postoperative insomnia and follicle-stimulating hormone (FSH) levels are independent risk factors for CPSP at 3 and 6-month postoperatively, with postoperative insomnia increasing risk and higher FSH levels exerting a protective effect. A higher BMI is a protective factor for 3-month CPSP but not significant at 6-month. Early identification and management of postoperative insomnia, along with preoperative FSH screening, may help reduce CPSP risk. This study provides a scientific basis for assessing and managing chronic postsurgical pain in breast cancer patients, with important clinical implications.

## Data Availability

The data that support the findings of this study are available from the corresponding author, upon reasonable request.
